# Personalized breast cancer onset prediction from lifestyle and health history information

**DOI:** 10.1371/journal.pone.0279174

**Published:** 2022-12-19

**Authors:** Shi-ang Qi, Neeraj Kumar, Jian-Yi Xu, Jaykumar Patel, Sambasivarao Damaraju, Grace Shen-Tu, Russell Greiner

**Affiliations:** 1 Department of Computing Science, University of Alberta, Edmonton, Alberta, Canada; 2 Alberta Machine Intelligence Institute, Edmonton, Alberta, Canada; 3 Alberta’s Tomorrow Project, Cancer Care Alberta, Alberta Health Services, Calgary, Alberta, Canada; 4 Department of Laboratory Medicine and Pathology, University of Alberta, Edmonton, Alberta, Canada; Aminu Kano Teaching Hospital, NIGERIA

## Abstract

We propose a method to predict when a woman will develop breast cancer (BCa) from her lifestyle and health history features. To address this objective, we use data from the Alberta’s Tomorrow Project of 18,288 women to train Individual Survival Distribution (ISD) models to predict an individual’s Breast-Cancer-Onset (BCaO) probability curve. We show that our three-step approach–(1) filling missing data with multiple imputations by chained equations, followed by (2) feature selection with the multivariate Cox method, and finally, (3) using MTLR to learn an ISD model–produced the model with the smallest L1-Hinge loss among all calibrated models with comparable C-index. We also identified 7 actionable lifestyle features that a woman can modify and illustrate how this model can predict the quantitative effects of those changes–suggesting how much each will potentially extend her BCa-free time. We anticipate this approach could be used to identify appropriate interventions for individuals with a higher likelihood of developing BCa in their lifetime.

## 1. Introduction

Breast Cancer (BCa) is the most diagnosed malignancy among women worldwide, with 2.26 million new cases diagnosed in 2020 [1, 2]. It accounted for 30% of estimated new cancer cases in American women in 2021 [3], and has a mortality-to-incidence ratio of 15% [4]. Researchers have mostly looked through the lens of the human genome to diagnose, prevent, and treat cancer [5, 6]. However, studies of identical twins have shown that genes are not the only source of cancer [7]. Instead, a research shows that external factors, such as lifestyle and environment, also contribute greatly to cancer development [8]. Metabolomic data also shows that more than 90% of cancers are caused by environmental exposures [9]. The identification of these external factors can benefit individuals as it can provide individuals with a “prescription” on how changing their lifestyle can reduce cancer risk.

Several recent observational studies have reported the impact of lifestyle and environment on the incidence of BCa. The incidence of BCa varies across continents and countries (27 per 100,000 in Africa and East Asia, but 97 per 100,000 in North America), reflecting the possible association between the risk of BCa development and local economic status, social and lifestyle factors [10]. One of the most significant cohort studies, the Million Women Study, confirmed the deleterious effect of hormone replacement therapy on BCa from over one million women [11, 12]. A study cohort of female BCa patients in Sweden showed that a large proportion of BCa cases might also be associated with pregnancy-related factors, hormone therapy, lifestyle factors (such as body mass index, exercise, alcohol consumption, diet habits, smoking, etc.), and other risk factors [13]. Other studies concluded that more than a third of breast cancers seem to be preventable through lifestyle changes in high-income countries [8]. In addition, there is controversy as to whether hormonal contraceptives increase the risk of BCa. Some studies believe that the associated effect is minimal [13], while others believe that long-term usage has a certain deleterious effect on BCa [14, 15].

Time-to-event (aka survival) analysis methods can greatly assist with building “disease onset” predictive models using modifiable BCa risk factors. This onset information can also help reduce BCa mortality and aid in women’s access to high-quality prevention, early detection, and treatment services [16, 17]. The famous Gail model/Breast Cancer Risk Assessment Tool [18, 19] assesses the BCa risk over the next 5 years using medical history information. The Breast Cancer Surveillance Consortium model [20] performed risk analysis to explore the relationship between age, race, family cancer history, and breast density to their risk of BCaO. However, all the above research fails to consider external factors, e.g., lifestyle and environment, in their risk factor analysis. The closest study as our might be the Nurses’ Health Study (NHS) [21, 22] and California Teachers Study (CTS) [23], which both include medical history information, postmenopausal hormones, and alcohol assumption to predict the BCaO risks. However, our study aimed to identify modifiable lifestyle factors by including a richer lifestyle trait, including dietary habits, physical activity information, hormone usage, and social support index, which the NHS and CTS studies were silenced on. Furthermore, in contrast to all these traditional risk models, few (if any) survival prediction models can use health history and lifestyle factors to calculate the probability curve of a woman developing BCa over time (rather than a single risk score).

An accurate estimation of the time to breast cancer onset (BCaO) in a woman based on her health history and lifestyle information can help suggest timely lifestyle changes to potentially delay her BCaO. This accurate personalized BCaO detection can thus improve the overall quality of life and reduce the burden of cancer treatment for a patient. This paper focuses on survival prediction models that compute time to BCaO for women at the individual level. We consider the following types of survival models to provide a context for our personalized approach to BCaO prediction [24]:

- **Individual Survival Distribution Models** produce a survival distribution (a probability curve for all future time points) specific to each individual.
- **Population-Level Survival Models** produce a survival curve for a group of individuals, e.g., the Kaplan-Meier estimator [25].
- **Single-time Probability Models** compute one survival estimate at a specific time for a particular individual. For example, the Gail model [18, 19] calculates the probability of breast cancer onset at 5 years from recruitment.
- **Time-Invariant Risk Models** produce a time-invariant risk score for each individual, such as the Cox proportional hazards model [26].

**Fig 1:**
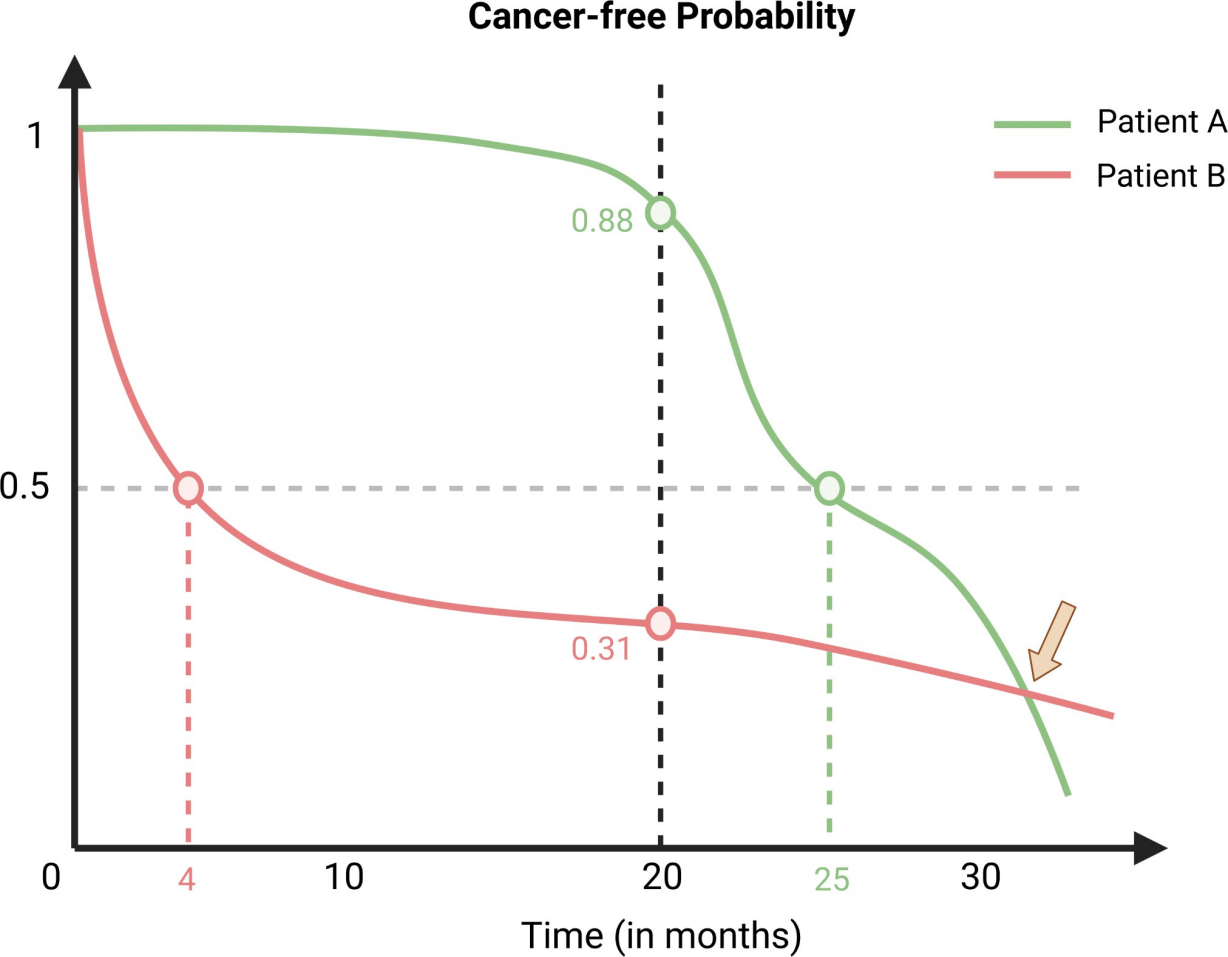
ISDs of two example participants–Patient A in green and B in red. Note that the ISD provides a person’s probability of being cancer-free, for each future time *t*, and the BCaO probability at time *t* is (*1—cancer-free-probability(t)*). Some useful statistics that can be computed from ISDs: the intersection of a patient’s ISD with the median probability line (the horizontal line at 0.5) is the median BCaO time for that patient; the intersection of an ISD with the vertical line at a target time (e.g., at 20 months) provides the cancer-free probability at that specific time-point. Note that, in general, multiple ISDs are allowed to crossover (shown with an arrow) as each patient has her own ISD computed from her specific features (unless a model assumes proportional hazards).

We focus on the first type of models that compute ISDs in this paper as other models do not provide several probabilities of BCaO over all future time points as required for our analysis. It should be noted that we use the term ISD to refer to the cancer-free probability curves in this paper (see examples in Fig 1). Fig 1 shows that Patient A’s BCaO is 88% at 20 months, this means our model predicts that there is an 88% chance that she will be BCa-free throughout those 20 months and a 12% chance that she will develop BCa in this time. ISDs have many desired properties for clinical applications–e.g., an ISD can provide personalized BCaO probabilities at any future time-point and can also be used to compute the expected time of BCaO, as well as confidence intervals around any such prediction. Fig 1 shows the ISDs of two example patients and highlights the important patient-specific statistics that can be computed from these ISDs.

Thus, our objective is to produce a tool that can accurately estimate the time until BCaO for each woman from her ISD, which is computed using her personal values of various features–including modifiable lifestyle features and health history information. We also propose that some of the modifiable lifestyle features could be used to suggest meaningful interventions to an individual to potentially delay BCaO, which we hope will eventually lead to a better quality of life (see Discussion section). The major strengths of this study are listed below:

- Curating a BCaO dataset, ATP-BCa, with Alberta’s Tomorrow Project (ATP) cohort that describes the relevant characteristics of 18,288 female residents in Alberta, Canada, and their corresponding BCaO times or censoring times. We believe this is the first time-to-event analysis dataset that contains the lifestyle and environmental features for making personalized BCaO predictions.
- Building ISD models from this ATP-BCa dataset that can predict individual BCaO from health history and lifestyle factors. We present a three-step approach (missing value imputation, feature selection, and ISD model) using a large observational dataset to develop an improved prognostic model for predicting BCaO time for healthy women. Note this is deviating from other existing models as we recruited only healthy adults without a previous history of cancer while most traditional risk models include BCa patients and healthy controls, which reflects the real-world scenario and hence more applicable for application. In addition, our model predicts BCaO time for healthy women instead of survival time for cancer-diagnosed patients.
- Identifying the important features for BCaO prediction. While our models include intrinsic (i.e., non-modifiable) features, we focus on actionable lifestyle features that participants can modify to potentially prolong their BCa-free time. These important actionable lifestyle features include features from supplement consumption (selenium and Vitamin E intake), social support index (connection and share index), and healthy food consumption (orange vegetable, fish, and whole-grain consumption). We also demonstrate how clinicians can use counterfactual results obtained from a participant’s ISD to provide them with actionable “recommendations” to potentially delay a woman’s BCaO.

## 2. Methods

### 2.1. Ethics approval and consent to participates

All data used in this study were de-identified before releasing to authors for analyses. Data access and analyses of this study complied with the provincial Health Information Act (HIA) in Alberta and Alberta Health Services (AHS) data access procedures and data disclosure guidelines. The study was approved by the local Health Research Ethics Board of Alberta (HREBA)—Cancer Committee under the protocol #-19-0188.

The secondary use of data in this publication was originally collected from participants through the Alberta’s Tomorrow Project [27, 28]. This data collection was reviewed and approved by the Health Research Ethics Board of Alberta (HREBA.CC-17-0461). More specifically, each participant signed a consent form, with a witness signature. In the event where a physical consent form was not signed, an implied consent was documented–e.g., if individuals participated in the telephone screen interview, they agreed to receive a mailed information package and returned a completed baseline questionnaire. ATP documents consents both electronically (through e-collection or scanning paper copies of the signed consent) and by paper (not yet scanned).

### 2.2. Dataset curation and description

**Fig 2:**
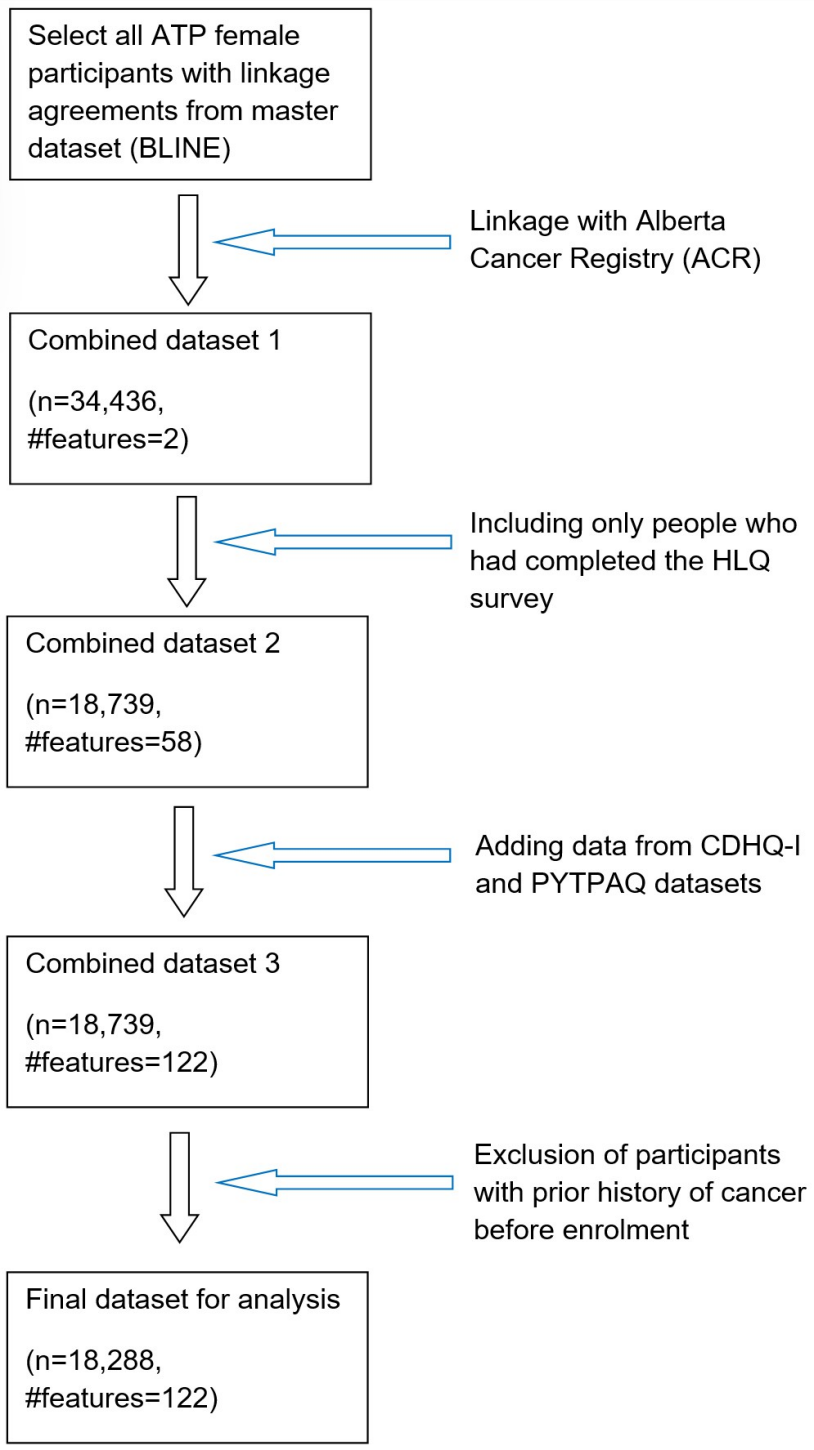
Scheme of data curation.

ATP was launched in 2000 to develop a deeper understanding of the etiological basis of cancers and other chronic diseases, to help prevent or reduce the incidences in the near future [27, 28]. This cohort recruited adult Alberta residents from 2000 to 2008, then regular follow-up these participants to collect lifestyle and health information. We selected women who filled out the Health and Lifestyle Questionnaire (HLQ) at the enrollment stage, as shown in the data assembly process in Fig 2. ATP-BCa also collected these participants’ diet and physical activity information through the Canadian Diet History Questionnaire I (CDHQ-I) and Past-Year Total Physical Activity Questionnaire (PYTPAQ). These participants were linked to the Alberta Cancer Registry (ACR) via their Personal Health Numbers (PHN) to collect detailed information on BCa diagnosis and stage. This study cohort, hereafter called “ATP-BCa”, includes 122 features (and time-to-event outcomes, e.g., censor indicator *δ* and time *T*) from 18,288 female ATP participants. Table 1 describes the characteristics of the uncensored and censored participants for this dataset. Please refer to [27, 28] for more details and patient characteristics in the ATP cohort.

**Table 1:** The definition and characteristics of female participants in the ATP-BCa dataset.

	Uncensored Participants	Censored Participants	Total Participants
**Event of Interest**	Breast Cancer Onset		
**Criteria for Breast Cancer Onset**	1. Ductal Carcinoma In-situ, Intraepithelial, Non-Infiltrating, Non-invasive;		
	or		
	2. Malignant or Primary Tumor		
**Start Time Definition**	Recruitment Month		
**Time-to-Event or Censoring**	Breast Cancer Onset Month	Last Follow-up Month ^a^	Breast Cancer Onset Month / Last Follow-up Month
**Number of Instances (%)**	605 (3.31%)	17683 (96.69%)	18288 (100%)
**Number of features (Number of Actionable Lifestyle features)**	122 (98)		
**Minimum–Maximum Age in Years**	35.22–70.18	35.08–70.34	35.08–70.34
**Mean Age ± Standard Deviation**	53.89 ± 9.23	50.47 ± 9.19	50.58 ± 9.21
**Maximum Follow-up Time (Months)**	201	207	207
**Median Follow-up Time (Months)**	86.88	160.68	106.20

^a^ The last follow-up time is the date of the last linkage with the Alberta Cancer Registry.

**Fig 3:**
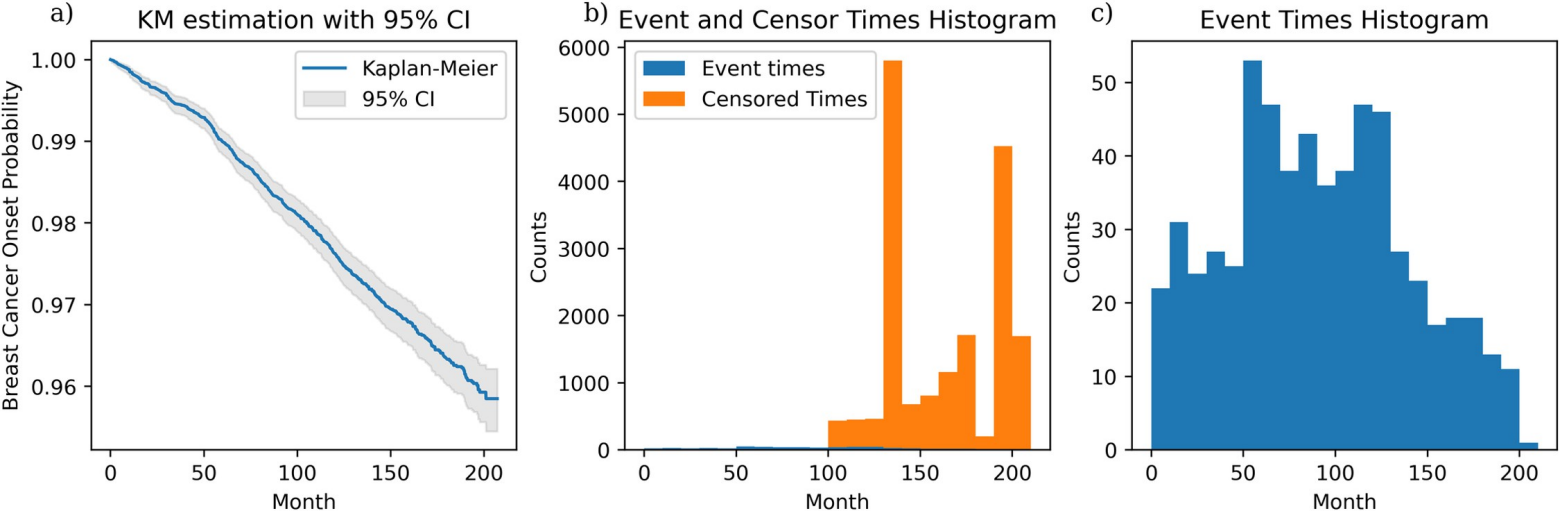
Kaplan-Meier estimation of the ATP-BCa dataset and the uncensored/censored time histograms. **(a)** Kaplan-Meier estimation with 95% confidence interval. Note that the y-axis starts from 0.95; **(b)** Uncensored (blue) and censored time (yellow) histogram of the participants in the ATP-BCa dataset; **(c)** Rescaling, and showing only the uncensored time histogram of the ATP-BCa dataset.

Fig 3 shows the cancer-free time characteristics for the ATP-BCa dataset. The Kaplan-Meier curves of Fig 3A show that most participants are not expected to develop BCa within 207 months, as the cancer-free probability for this time is 95.85%, with a 95% confidence interval of 95.44% to 96.22% (computed using Greenwood’s formula [29])–this confidence interval strengthens the fact that 96% of the individuals did not experience BCaO in the ATP-BCa dataset. Fig 3B presents the event and censor time histogram of all 18k participants in the ATP-BCa study, showing that the first censored participant appears at the 95^th^ month after she was recruited. Thus, the personalized BCaO model built from the ATP-BCa data should incorporate a large number of censored patients with censor times between 95 to 207 months Fig 3C shows a zoomed-in version of the histogram of event times as these are occluded in Fig 3B. Note that we consider a broader definition of BCaO that includes ductal carcinoma in-situ (DCIS) because (1) DCIS is often considered the earliest form of breast cancer, and since our goal was early BCaO detection, it is reasonable to use DCIS as cancer onset event; (2) DCIS has a potential to develop as an invasive carcinoma, which means that the patients with DCIS are at a higher risk of having invasive breast cancer incidence.

### 2.3. Feature categories

The dataset has rich feature sets including baseline information (BLINE), HLQ, CDHQ-I, and PYTPAQ [27, 28]. As mentioned in the introduction, we want to determine the lifestyle risk factors associated with BCaO, focusing on features that participants can alter or modify in the hope of delaying onset. Below we summarize the feature fields and define which sets of variables our experts consider actionable lifestyle factors.

- **BLINE (2 features):** There are just two baseline variables: age and geographic (rural or urban) characteristics; obviously, age is not an actionable factor while the geographic location is actionable.
- **HLQ (56 features):** This feature set contains the participant’s self-reporting answers about personal and family medical history, history of cancer screening and family history of BCa or other cancers, reproductive health, smoking, social support, anthropometric measurements, and demographic characteristics. This HLQ questionnaire was acquired at the recruitment time for participants. While the participant has the option of changing her future lifestyle (related to smoking, social support, and anthropometrics), she of course cannot modify previous features, such as her medical history, cancer screening behavior, reproductive health, nor demographic characteristics.
- **CDHQ-I (52 features):** This feature set contains self-reporting food and nutrient intakes information in the preceding year of subjects’ enrolment in the project–including daily consumption of alcohol, dairy, meat and vegetable, vitamin intake from supplements, etc. All CDHQ-I features are considered actionable lifestyle factors.
- **PYTPAQ (12 features):** This feature set describes the type and average amount of physical activity in the year before that subject enrolled. It considers four types of activities: job-related physical activity, household-related physical activity, leisure-time physical activity, and transportation-related physical activity. All PYTPAQ features are considered actionable lifestyle factors.

We distinguish each feature by whether it is actionable or intrinsic, as described in S4 Table in the Supplementary file. We treated 24 features as intrinsic, while the remaining 98 as actionable.

### 2.4. Preprocessing

The raw ATP-BCa dataset is missing some values due to two reasons: (1) whole sections of questionnaires are missing for participants who were part of different sub-studies and so, for example, a participant was instructed to complete the HLQ but not the CDHQ-I or PYTPAQ questionnaire; or (2) specific variables are missing, for example, BMI information is not available due to missing height or weight data for some participants. Then we experimented with three different data imputation methods to fill out the missing values for the remaining features:

- **Median Value Imputation (MVI)** is a univariate non-parametric method that fills the missing values with the median value of that feature.
- **K-Nearest Neighbors (KNN)** Imputation is a multivariate non-parametric method for imputation: If participant *p* is missing the value of feature *f*, this method first finds *p*’s *k* “nearest neighbors”, based on Euclidean distance (after removing feature *f*, of course). We then set *p*’s “*f*” value to be the mean of the “*f*” value (after one-hot encoding categorical features) of those *k* nearest neighbors. Here we set *k = 2*, meaning we use the 2 closest neighbors of each participant to calculate the missing feature value.
- **Multiple Imputation by Chained Equations (MICE)** is a multivariate parametric method, which iteratively models each feature with missing values as a function of other non-missing features and then impute that estimate [30].

One difference between these three imputation methods is that MVI assumes the features are independent, while KNN (resp. MICE) computes the relevant value as a learned non-linear (resp., linear) function of the other feature values. After completing the missing value imputation, we apply feature normalization to non-categorical features and one-hot encoding to categorical features.

### 2.5. Feature selection

As discussed in Introduction section, identifying key actionable lifestyle features is important because this can help a patient delay her BCaO time by adopting a healthier lifestyle. However, clinicians will prefer to recommend a few (instead of hundreds) most important actionable features that a patient can modify to possibly reduce her BCaO probability by (say) 10% at 10 years in the future. With this in mind, we did a thorough feature selection analysis of the whole ATP-BCa dataset with 122 available features. This section briefly introduces the feature selection methods and the extraction of the features related to BCaO. Details of the implementation and the comparison are available in Results section.

- **Univariate Cox** fits a Cox proportional hazard (CoxPH) model using one feature at a time and uses the Wald test to assess the significance of that feature [26, 31]. If the *p-*value of the Wald test is lower than a specified value (we used threshold = 0.001, as recommended in [32]), then the feature is considered significant and is retained in the dataset; otherwise, it is removed.
- **Recursive Feature Elimination (RFE)** is a standard feature selection method in classification and regression tasks [33]. In this study, we altered it to adapt to accommodate censored data by iteratively fitting a CoxPH model and removing the least important feature, until it retains only the desired number of features. We set the desired number of features to 10, which is comparable to the number of features selected by the above method.
- **Minimum Redundancy, Maximum Relevance (mRMR)** is a “minimal optimal” feature selection algorithm, meaning that it seeks to find a feature set that gives the best possible predictive performance, given a fixed number of features. Inspired from [34], we adapted the mRMR method to use the C-index to estimate the correlation between a variable and binary outcome of BCaO. We again set the desired number of features to 10 for mRMR.
- **Multivariate Cox** fits a linear CoxPH model using all features and uses an elastic net penalty for feature selection [35]. Features with non-zero weights are retained in the dataset by this method. The hyper-parameter (lasso/ridge ratio) selection is made through grid search based on validation performance computed as the C-index.

### 2.6. Evaluation metrics

This section briefly describes the metrics we used in this study for evaluating ISD models. We evaluate the model from the following three aspects: how close is the predicted BCaO time close to reality (L1-Hinge loss), pairwise ranking accuracy (Concordance index), and model calibration (D-calibration). For the simplicity, we only provide a plain description of these metrics, while a precise definition can be found in the section A in S1 Text. We prefer a model that has the lowest L1-Hinge loss among all D-calibrated models and use C-index to break L1-Hinge loss ties (see Discussion section).

**Fig 4:**
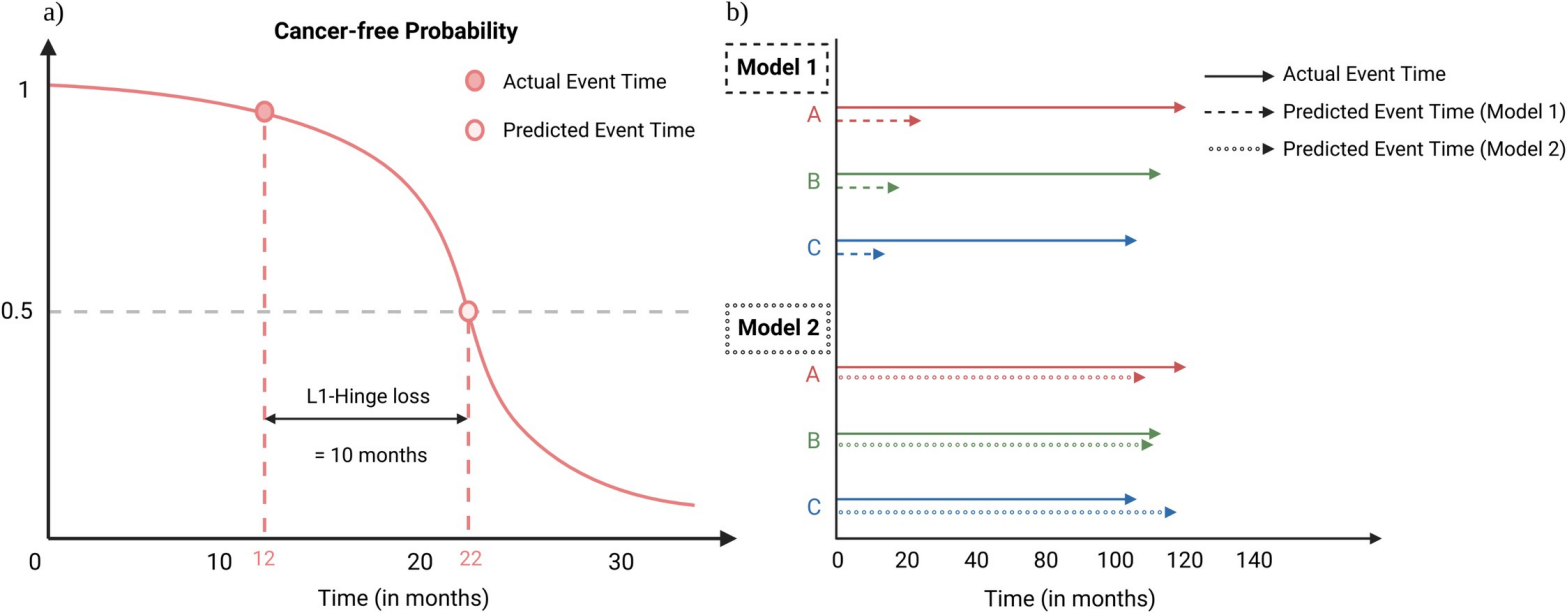
Illustration of the L1-Hinge loss and its comparison with the C-index. **(a)** L1-Hinge loss calculation from an ISD curve for an uncensored participant in ATP-BCa dataset. **(b)** Comparison of the L1-Hinge measurement and the C-index (see Section 5.3 for details). The dashed and dotted arrows indicate the expected BCaO times. (Note these examples are of uncensored individuals).

- **L1-Hinge loss** is like the mean absolute error (MAE) measurement in the regression task, with lower scores (lower bounded by 0) indicating better performance. Fig 4A illustrates the L1-Hinge loss calculation, which is the absolute difference between actual and model-predicted BCaO times (which is the median of the person’s ISD). It incorporates censoring by assigning 0 loss to any patients whose predicted event times are later than the censoring times, and the loss of *[censoring time—predicted BCaO time]* if the predicted BCaO times are earlier than the respective participants censoring times. For formal definitions and details, please see section A.1 in S1 Text.
- **Concordance index (C-index)** is one of the most popular metrics for evaluating survival prediction models, with higher scores (upper bounded by 1) indicating better performance. It checks if the predicted order of events matches the true order for every “comparable” pair in a dataset. C-index includes censored instances into its calculation by appropriately defining the “comparable” pairs. A pair is “comparable” if we can determine who has the BCaO first. Finally, the C-index is calculated as the percentage of concordant pairs (correctly ordered predicted event times) among all comparable pairs. Please see section A.2 in S1 Text for details.
- **Distribution calibration (D-calibration)** is a statistical test that assesses whether the probability prediction provided by an ISD curve is reliable [24]. Note that the popular measures calibration assessment such as calibration regression plots, *1-calibration*, etc., are not applicable to ISD models because the ISD models provide event probabilities at all future time-points instead of just one event probability or risk value at a fixed time-point. D-calibration splits the probability-axis of an ISD into *B* equal-sized bins (we used *B = 10* in this paper) and assess if the actual number of events within each probability bin is statistically similar to the model-predicted number of events, claiming a model is D-calibrated if the *p*-value from Hosmer-Lemeshow test is > 0.05 [24]. For formal definition of D-calibration calculation and how to handle censored patients, please see section A.3 in S1 Text for details.

### 2.7. Experimental design

The primary outcome for this study is the prediction of BCaO probabilities within the next 207 months (maximum follow-up time in currently available ATP-BCa dataset) given the participant does not have BCa at recruitment. We then compared the effectiveness of nine survival algorithms, ranging from traditional statistical models to deep learning models: Cox proportional hazard model (CoxPH) [26], CoxPH model with elastic net penalty (CoxNet) [35], accelerated failure time (AFT) [36], random survival forest (RSF) [37], gradient boosting Cox machine (GBCM) [38], component-wise gradient boosting Cox machine (CW-GBCM) [39], DeepHit [40], deep survival machine (DSM) [41], and multi-task logistic regression (MTLR) [42, 43].

We applied the same experimental procedure to all models using the ATP-BCa dataset as input:

- We divided the ATP-BCa dataset into 5 cross-validation (5CV) splits, stratified for both censor indicator, *δ*, and BCaO time, *T*.
- The imputation and feature selection process were performed after the 5CV dataset.
- When necessary, we ran internal 5CV, within the training set, for grid-search-based hyperparameter selection (details appear in the Supplementary material), seeking the best (in terms of the lowest L1-Hinge loss or highest C-index, depending on the model) hyperparameter settings.
- Discrimination measurements (L1-Hinge for individual-level prediction, C-index for pair-wise-level prediction) and calibration measurement are reported on the predicted BCaO probability curves of each ISD model. We do not report the Brier Score (nor Integrated Brier Score), because the high censoring rate for the ATP-BCa dataset, means these measure have limited clinical utility [44].
- We also propose a soft-L1-Hinge loss as the objective function and show that directly minimizing this objective function for several epochs after pre-training the original model results in better performance. We compare the training loss for this approach, versus others; see the Supplementary material. The Discussion section presents the details of these evaluation metrics and their limitations in the clinical application in.

The above procedure is visually demonstrated in S1 Fig.

## 3. Results

In this section, we report the results of our experiments that compare different imputation methods, different feature selection methods, and different ISD methods for BCaO prediction in ATP-BCa dataset. Specifically, we experimented with three imputation methods–median value imputation (MVI), K-Nearest Neighbor (KNN), and Multiple Imputation by Chained Equations (MICE); four feature selection methods–univariate Cox feature selection, Recursive Feature Elimination (RFE), Minimum Redundancy Maximum Relevance (mRMR), and multivariate Cox feature selection; and several ISD models including Cox proportional hazard model (CoxPH) [26], CoxPH model with elastic net penalty (CoxNet) [35], accelerated failure time (AFT) [36], random survival forest (RSF) [37], gradient boosting Cox machine (GBCM) [38], component-wise gradient boosting Cox machine (CW-GBCM) [39], DeepHit [40], deep survival machine (DSM) [41], and multi-task logistic regression (MTLR) [42, 43].

We report the L1-Hinge loss, D-calibration, and time-invariant concordance index (see Discussion section and section A in S1 Text for detailed explanation) as described [24]. Note that the time-invariant concordance index is different from the standard single-time concordance in a fundamental way: the former measures the concordance on the scope of event times while the latter focuses on the single time probabilities. All reported results are the average (± standard deviation) of the five folds cross-validation. We prefer D-calibrated models with the smallest L1-Hinge loss and use C-index to break L1-Hinge loss ties (see Discussion section). The best performing combination in our experiments was the MTLR model with MICE imputation and multivariate Cox feature selection, which was D-calibrated and had an average L1-Hinge loss of 7.173 months (smallest among all D-calibrated models).

**Fig 5:**
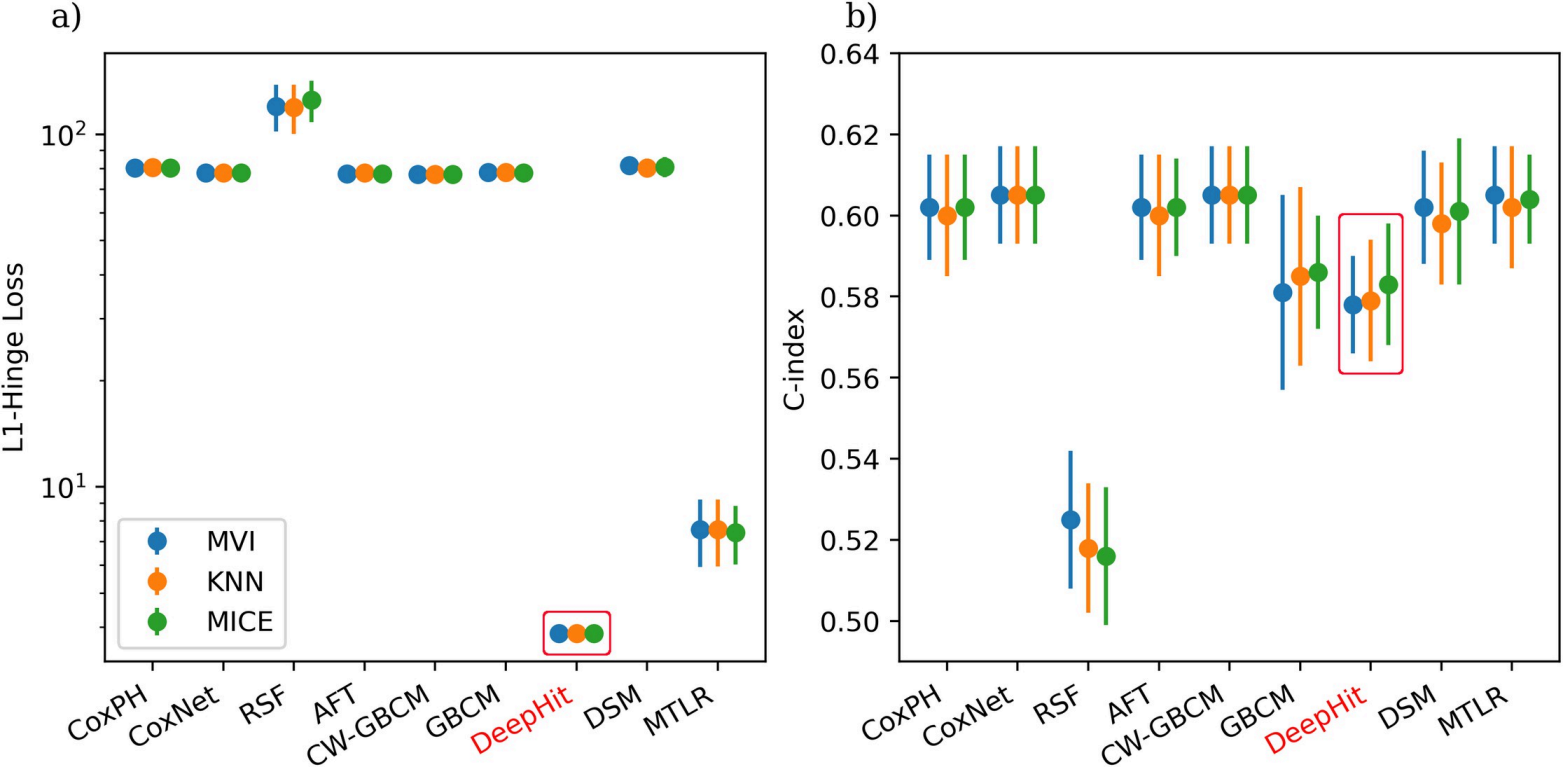
Imputation method comparison using multivariate Cox feature selection method. The red rectangle shows the non-calibrated models.

**Fig 6:**
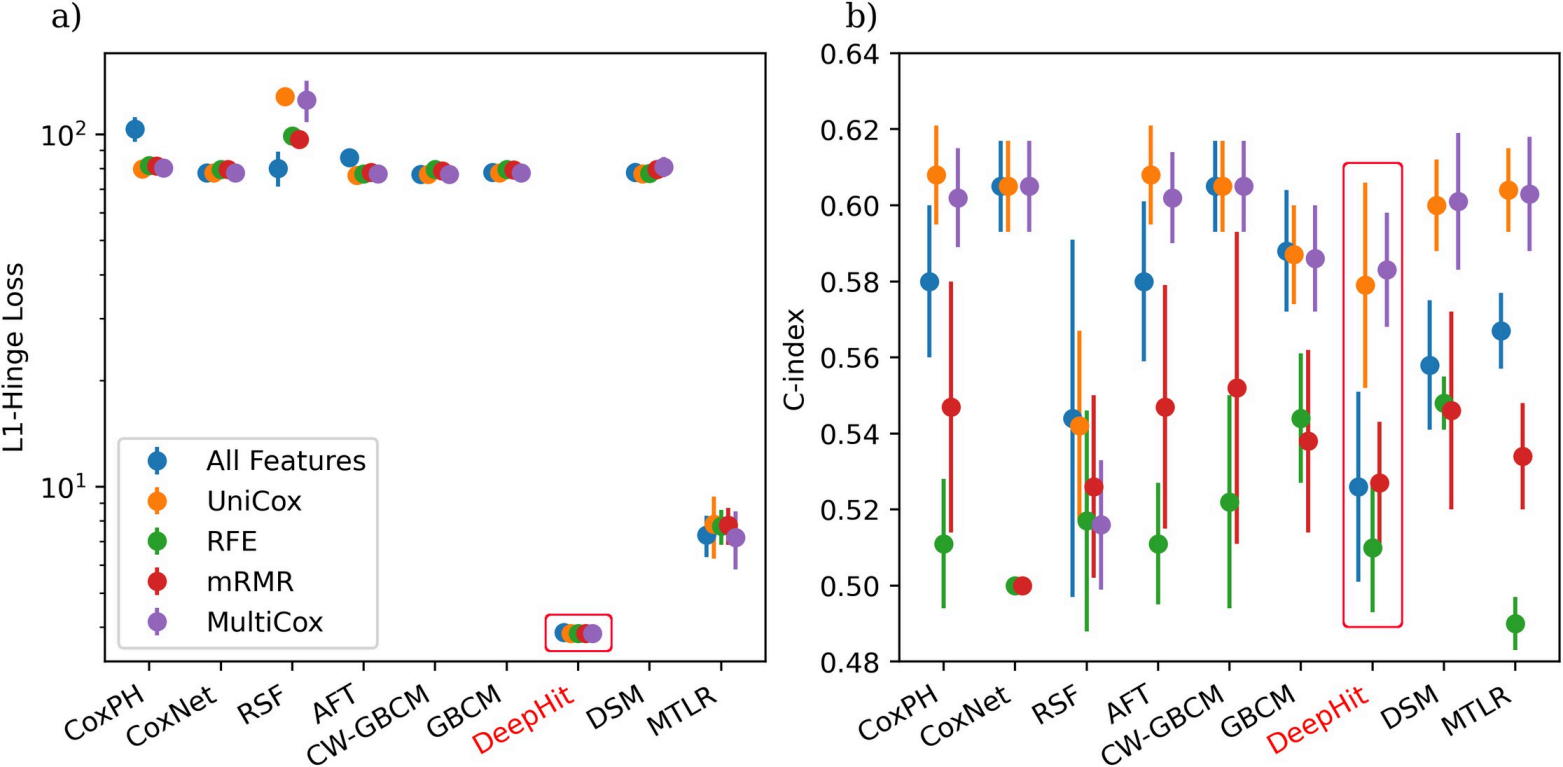
Feature selection method comparison using MICE imputation. The red rectangle boxes represent the non-calibrated models.

The comprehensive results are presented in S3 Table of the Supplementary material. Due to the space constraints in the main text, we only present the comparison of (a) imputation methods with the fixed (best) feature selection method (Fig 5), (b) feature selection methods with fixed feature imputation method (Fig 6), and (c) ISD models with fixed feature imputation and feature selection methods (Table 2), respectively.

**Table 2:** Comparison of all ISD algorithms using MICE imputation and multivariate Cox feature selection.

MICE Imputation Multivariate Cox Feature Selection	L1-Hinge Loss (in months)		Concordance Index	
	Mean	Standard Deviation	Mean	Standard Deviation
**CoxPH**	80.286	2.523	0.602	0.013
**CoxNet**	77.653	0.707	0.605	0.012
**RSF**	125.086	16.731	0.516	0.017
**AFT**	77.261	2.594	0.602	0.012
**CW-GBCM**	77.122	0.879	0.605	0.012
**GBCM**	77.763	0.738	0.586	0.014
DeepHit*	3.841*	0.018*	0.583*	0.015*
**DSM**	80.887	5.356	0.601	0.018
**MTLR**	**7.173**	**1.341**	**0.603**	**0.015**

* Not D-Calibrated model.

The comparison of three data imputation methods, with the fixed feature selection method, is shown in Fig 5 and S3 Table in the Supplementary material. All models in this comparison used the unified multivariate Cox feature selection method. The red highlight on “DeepHit”, in both Fig 5 and S3 Table, means that the DeepHit models are not D-calibrated (see Materials and Methods section), while all other models are D-calibrated. These results demonstrate negligible difference between three imputation methods, however, MICE imputation produced models with relatively lower average L1-hinge loss (Fig 5A), and MVI and MICE imputation both obtained higher C-index than KNN imputation (Fig 5B). Recall that a lower L1-hinge loss indicates better performance while a higher C-index indicates better performance.

The feature selection method comparison appears in Fig 6 and S4 Table in the Supplementary material. All models in this comparison used the MICE imputation method. The red rectangle boxes in Fig 6 show the non-calibrated models (all DeepHit models). Fig 6A shows that univariate Cox and multivariate Cox methods obtain relatively lower L1-hinge losses and higher C-index in most models. The best performance is obtained using the MICE imputation method with Multivariate Cox feature selection using the MTLR model, as it has the lowest L1-hinge loss among all D-calibrated models and has a competitive C-index score. We can see that implementing feature selection (either with univariate or multivariate Cox) significantly boosted the performance for all ISD models. The poor performance of the models that used all features as input might be due to potential overfitting on the large set of features. However, RSF models without feature selection showed an obvious performance boost (mostly for L1-Hinge loss), which is probably because RSF has its own internal feature selection mechanism and thus does not require another external feature selection method.

Table 2 demonstrates the models’ performance on the ATP-BCa dataset with the best imputation method (MICE) and the best feature selection methods (multivariate Cox). We see that both discrete-time models–MTLR and DeepHit–have relatively smaller L1-hinge loss values, which may be because discrete-time models do not require the strong assumptions (distribution nor proportional hazard) that are required by other continuous-time models. The MTLR model also has the second-best C-index value among these nine models.

While DeepHit does have the lowest L1-hinge loss, this is not the best model, for three reasons: (1) DeepHit is the only model that is not D-calibrated (see S2 Fig in the Supplementary materials), which means its probability estimations at individuals’ BCaO times are not reliable and hence, not acceptable for clinical usage [45, 46]; (2) DeepHit requires that all BCaO probability curves must reach 0 at the last observed time, which implies that everyone must have BCaO before the maximum time in the dataset (207 months for ATP-BCa), which is contrary to the ATP-BCa dataset described in Results section; (3) DeepHit has smaller C-index than the other models, which means it cannot reliably rank the priority of pairs of patients, etc.

## 4. Discussion

### 4.1. Selected actionable lifestyle factors

In the past decades, many clinical studies have used statistical or machine learning methods to identify risk factors that reliably predict BCa development. However, most of the studies focused on intrinsic risk factors such as hereditary factors [5, 6], hormones and metabolic molecules [14, 47], as well as reproductive factors [48, 49]. In this study, we expand the scope of factors to include both those intrinsic (hereditary, hormone, reproductive) as well as lifestyle factors. We then consider four different feature selection methods for selecting the most important factors. Experimental results (see Results section) indicate that the multivariate Cox feature selection method (with MTLR) has the best performance (smallest L1-hinge loss among all D-calibrated models). Table 3 shows the features selected by applying the multivariate Cox feature selection to the entire ATP-BCa dataset (after pre-processing and MICE imputation; see Materials and Methods section).

**Table 3:** Important features selected by multivariate Cox feature selection.

Feature selected	Plain description	Coefficient	Type
BLINE_AGE_AT_BASELINE	Age at recruitment	0.3007	Intrinsic
CDHQ1_SELENIUM_SPL	Selenium intake	-0.0595	Actionable
HLQ_SPT_11	Connection index with Family and Friends	0.0194	Actionable
CDHQ1_ORANGE_VEG_MYP	Orange vegetable consumption	-0.0184	Actionable
CDHQ1_FISH_HI_MYP	Fish with high Omega-3 consumption	-0.0148	Actionable
HLQ_FRH_1	Age of first menstrual period	-0.0117	Intrinsic
HLQ_SPT_16	Share index with Family and Friends	0.0094	Actionable
HLQ_FRH_25	Hormone replacement (usage) history	0.0055	Intrinsic ^a^
CDHQ1_WHOLE_GRAIN_MYP	Whole-grain consumption	0.0052	Actionable
CDHQ1_VITAMIN_E_SPL	Vitamin E intake	0.0010	Actionable

^a^ Note that we consider hormone replacement history as an intrinsic feature from the patients’ perspective as they cannot ask for treatment on their own. However, it could be considered as actionable from the clinicians’ perspective.

Table 3 divides the selected features into two types: intrinsic and actionable. A feature is “actionable” if it can be modified–such as diet or exercise. By contrast, a feature is “intrinsic” if it cannot be changed–e.g., current age, age of first menstrual period, and hormone usage history. The positive coefficient values of age and hormone usage history (in Table 3) indicate that an increase in the values of these features increases the likelihood of BCaO. Similarly, the negative coefficient values of the first menstrual period information indicate that women with late first menstrual period have reduced BCaO probability. These findings are consistent with the results reported in previous studies [14, 15, 48–50].

Below we discuss the selected actionable features from three aspects: supplement consumption, social environment index, and healthy food consumption.

#### 4.1.1. Supplement consumption (Selenium intake and Vitamin E intake)

Selenium intake from supplements is identified as the second most important feature and first important feature among the actionable features. Its negative coefficient value in Table 3 means larger values suggest smaller BCaO–i.e., earlier onset of BCa. However, retrospective research regarding selenium intake and human BCa development is limited. Cann et al. [51] suggested a preventive role of selenium in BCa, which was assessed from the association between dietary preferences of the U.S. and Japanese women and their relatively lower incidence rates of BCa. Although there is evidence that selenium has a preventive effect on human BCa from the study of dietary intakes and whole blood selenium levels [52], rigorous retrospective and prospective studies are still needed to confirm these hypotheses.

Additionally, there is evidence that metabolic selenium is associated with BCa incidence. A meta-analysis-based random effect analysis supports an inverse association between selenium concentration in serum and BCa risk [53]. A recent cohort study of 2295 Sweden patients also concluded that the combination of high serum iodine levels and high selenium levels was associated with a lower risk of BCa [54]. However, some researchers suggest that alterations in serum concentrations of selenium in women with BCa appear to be a consequence, rather than a cause of cancer [55].

The small positive coefficient value for Vitamin E intake suggests that a higher intake of this vitamin might increase the likelihood of BCa. Although the relationship between Vitamin E and cancer development is not yet established, some researchers suggest that Vitamin E may exert growth inhibitory effects on cancer cells, which may indicate that Vitamin E can reduce the tumor burden [56, 57]. However, our findings suggest that Vitamin E might increase the likelihood of BCaO in ATP participants, the bioavailability of Vitamin E from supplements is unclear [58]. Vitamin E is hydrophobic and bulk of this vitamin partitions into the adipose tissue (body fat) [59, 60] and hence, more research is required to find the actual association of Vitamin E intake, bioavailability and its association with BCaO.

Except for these direct causal influences, we hypothesize that Selenium and Vitamin E intake from supplements would also have an indirect association with the incidence of BCa. It might be due to some unobserved confounders that have a direct effect on both selenium/Vitamin E intake volume and BCa incidence. For example, women who pay more attention to personal health may choose a healthier lifestyle (e.g., smoke and drink less, exercise regularly, and maintain a healthy diet) and take their required supplements regularly. A healthy lifestyle, in turn, will play a direct role in preventing BCa, and selenium and Vitamin E intake may only have some (non-causal) correlation. Finally, both selenium and Vitamin E intake were self-reported in the ATP-BCa dataset, and thus, we need an objective quantification of these variables to accurately assess their relevance for BCaO prediction.

#### 4.1.2. Social support (connection and share index with family and friends)

Social support is a well-recognized determinant in personal health [61]. Although prospective research has shown that socially isolated individuals have a physiological milieu that promotes tumor growth [62, 63], at present, there are no reported quantitative research on the impact of social environment and social isolation on cancer. Our early-stage research dataset, ATP-BCa, is the first attempt to transform the social environment into 20 semi-quantitative indicators. Table 3 shows that the multivariate Cox feature selection method has identified HLQ_SPT_11 (“Connection index with family and friends”) and HLQ_SPT_16 (“Share index with family and friends”) as important features for predicting BCaO. Surprisingly, these two features have positive coefficients, meaning they appear deleterious in the ATP-BCa dataset. This seems to differ from the previous research that suggests that more social interactions might lead to a better overall quality of life for breast cancer patients [62–64]. However, note that our study focuses on personalized prediction of BCaO and not on the quality of life assessment of breast cancer patients. It might be possible that some unobserved confounders lead to a higher BCaO rate as well as a higher social support index in the ATP-BCa dataset. For example, people with a high social support index might also tend to have higher alcohol consumption and irregular eating and sleeping timings (“party animal” lifestyle). Again, this field of quantitative research is still in its early stages, and further research is needed to confirm whether these findings can be used for intervention purposes.

#### 4.1.3. Healthy food consumption (orange vegetable, fish with high Omega-3, and whole grain)

Healthy eating habits are essential for good physical health and growth. In Table 3, orange vegetables (negative coefficient), fish with high omega-3 (negative coefficient), and whole-grain consumption (positive coefficient) are identified as the three most important features among all healthy foods. Some of these findings are consistent with the existing research results, as described below.

- **Orange vegetables:** Farvid et al. [65] used CoxPH to estimate hazard ratios with fruits and vegetable consumption as risk factors of BCa. Their research suggested that higher consumption of fruits and vegetables, especially cruciferous and yellow/orange vegetables, can reduce the risk of BCa [65]. In addition, it is well known that orange vegetables are rich in carotenoids, such as α-carotene, β-carotene, lutein, lycopene, etc. Many studies have demonstrated that a higher intake of these carotenoids is associated with a lower risk of pre- and postmenopausal BCa incidence [66, 67].
- **Fish with high Omega-3**: Omega-3 fatty acids have been demonstrated to have a protective effect against BCa incidence [68]. Kim et al. [69] used a multivariate logistic regression model in a 718-Korean-patient cohort study and concluded that a high intake of fatty fish was associated with a reduced risk for BCa in both pre- and postmenopausal women. Kaizer et al. compared the incidence of BCa with the estimated consumption of fish, other foods, and nutrients for women from different countries [70]. Their results showed that the percentage of fish consumption was negatively correlated with the incidence of BCa, which was consistent with the protective effect described above as well as with our findings.
- **Whole grain**: As part of a healthy diet instruction, studies have shown that eating more whole grains can reduce the risk of multiple diseases [71]. But its preventive effect on cancer, especially BCa, has yet to be confirmed. In a case-control study, it was observed that large amounts of whole grain foods were associated with a 0.4-fold lower likelihood in BCa risk compared with people who rarely consumed whole grains [72]. Moderate consumption of whole grain foods is associated with a 0.6-fold lower likelihood of the incidence of BCa [72]. Another case-control study also observed the same trend by investigating the pre-menopausal BCa likelihood [73]. The positive coefficient of whole grain intake in Table 3 also suggests that higher intake will increase the risk of BCa incidence. However, these findings should be confirmed with future rigorous research investigations in a case-control setting.

### 4.2. Interventions on lifestyle factors

After identifying the most important actionable features from the ATP-BCa dataset, both participants and doctors may have prognostic questions such as: How to quantify these actionable features? What actionable features should be changed to maximize a person’s cancer-free time (that is, how to delay or prevent BCaO)? How much can we delay a woman’s BCaO through interventions on actionable risk factors? This section will provide two examples of actual patients in the ATP-BCa dataset that suggest how one might use our ISD model for prognostic analysis. (While these examples are based on instances in our ATP-Bca dataset, we have made slight modifications to protect the privacy of the participants).

**Example 1.** Consider a female participant who was recruited at age 62 into the ATP-BCa study. Her regular follow-up revealed BCaO at the 197th month after her recruitment in the study. At recruitment, she had reported that she did not take any selenium supplement in the year proceeding to her recruitment day. Our learned model (MTRL + MICE imputation + multivariate Cox feature selection) predicted her BCaO time as 267 months (at 85 years of age) from the date of recruitment, using the features recorded at the time of her recruitment in the ATP-BCa dataset. Note that our predicted BCaO time is very close to her actual BCaO time: 267 months vs. 197 months with L1-Hinge loss of 70 months. In a counterfactual setting, we recomputed her BCaO time by changing her selenium intake to 47 unit/day (from earlier 0 unit/day), which is the maximum dose in the ATP-BCa dataset, but keeping the other risk factors unchanged; here, our model predicted that her counterfactual BCaO time would be 362 months (an increase of 95 months (approximately 8 years) from the previous estimate).

**Example 2.** Another ATP-BCa female participant was 37 years old at the time of recruitment. The periodic follow-up with linkage to ACR found that she was diagnosed with BCa after 390 months in the ATP-BCa study. In the CDHQ-I questionnaire, she reported that she rarely eats orange vegetables (0.1 cups/day), which include pumpkin, sweet potatoes, carrots, beef and chicken mixtures, and soups [74]. Our MTLR model predicted her BCaO time as 343 months (66 years old) if she maintains her current lifestyle. Suppose she increases orange vegetables to her daily meal equal to the average intake in the ATP-BCa dataset (0.21 cups/day). In that case, the predicted BCaO time goes up to 367 months (66 years old), which means we expect her to gain an additional 24 cancer-free months. If she increases orange vegetables to the maximum intake in the ATP-BCa dataset (2.31 cups/day), the predicted event time goes up to 660 months (92 years old), which gives her a predicted cancer-free time of 26 years.

### 4.3. L1-Hinge loss versus C-index

C-index is one of the most popular metrics, which has dominated the field of time-to-event prediction evaluation for decades. It focuses on measuring the pairwise discriminability of a survival model, with a larger value indicating a superior model performance. Intuitively, the C-index only assesses a model’s ability to correctly order the event-time of the patients. Although C-index is useful in several clinical problems that require such patient comparison, such as prioritizing patients for liver transplants [75], it is not an appropriate metric for evaluating patient-specific BCaO prediction models. This is because there is no need to predict correct patient order in BCaO prediction. Why will Ms. Smith be interested in knowing whether she will get BCa before some random stranger Ms. Jones? Also, a medical professional will administer care to his/her patient based on that patient’s specific risk of BCaO instead of providing recommendations based on the BCaO risk of another patient (although a clinician can use the ordered risk of patients to prioritize whom (s)he wants to see first).

Thus, Ms. Smith may want to know when she will likely develop BCa based on her current lifestyle. The physicians also want to know the predicted time of BCaO for Ms. Smith to decide whether to suggest appropriate cancer screening tests or provide her with an immediate treatment plan. To be able to answer these types of questions, we need a BCaO prediction model whose predicted BCaO time is close to the actual BCaO time for individuals in a given dataset–this is precisely quantified by the L1-Hinge loss metric [24]. Thus, we propose that L1-Hinge loss should be used to evaluate the performance of personalized BCaO prediction models.

It is tempting to believe if C-index and L1-Hinge loss prefer the same model–i.e., if Model 1 has a higher C-index than Model 2, then Model 1 must have a lower L1-Hinge loss, and vice-versa. However, this is not always the case. Fig 4B shows two ISD models that each predict BCaO times for three participants (A, B, and C) using their respective ISD curves. We see the actual time to BCaO is patient C followed by B and then A. Defining an ISD model’s risk score as (the negative of) the median value the ISD curve, we see that Model 1 has the perfect C-index (= 1) while its predicted BCaO times are far from the actual BCaO times (L1-Hinge loss ≈ 90 months). Model 2 has the worst possible C-index (= 0), but its BCaO time estimation is more accurate (L1-Hinge loss ≈ 10 months). This example demonstrates that C-index’s preferences do not match L1-Hinge loss’s, as these metrics measure different aspects of the model: We see that also a model with a perfect C-index can have very poor estimates at the individual level. Moreover, L1-Hinge loss focuses on measuring the accuracy of individual prediction, while C-index measures the accuracy of pairwise discrimination, which is often not relevant for personalized decision making in clinical settings as it is a relative performance measure that requires pairs of patients for its computation. Therefore, we prefer models that have the lowest L1-Hinge loss among all D-calibrated (*p*-value > 0.05) models.

### 4.4. Future directions

This early-stage work addresses the need for predicting the time of a woman’s BCaO from her medical history and lifestyle features. Our observational study included several experiments to assess various combinations of data imputation, feature selection, and ISD models for computing patient specific BCaO probability curves. We also provided guidelines for appropriate metrics for evaluating the BCaO models and discussed possible counterfactual usage of these personalized BCaO probability curves. We think that further research can address the following limitations of this paper: (1) The ATP-BCa dataset contains two categories of lifestyle features: diet-related and physical-activity-related features. While the multivariate Cox feature selection method selected 7 actionable features, none involve physical activities. This may be because the effects of the physical activity traits on BCaO have been indirectly revealed by the selected features. However, the direct effects of physical activity-related features still require further investigation. (2) Due to the nature of the dataset, all features were derived from patients’ responses to the self-reported questionnaires, which means those responses might not be objective and might contain information bias, and the range of questions might not be comprehensive. For example, Introduction section mentioned that a patient’s genetic features, the breast density features, and the features extracted from medical images might lead to more accurate personalized BCaO predictions. In addition, the current database has been dominated by female Caucasians; it would be useful to include people of other ethnicities. (3) Our analysis assumed that the patient’s medical history and lifestyle characteristics were stable. This implicit assumption runs throughout our study and specifically for our discussion in this section. However, in the real world, a patient’s medical and lifestyle characteristics do change over time, and some people can even change their lifestyle habits drastically in a short span of time. These changes need to be incorporated into the datasets and the analysis requires ISD models that could handle temporal-feature datasets.

## 5. Conclusion

This paper has demonstrated the effectiveness of learned individual survival distribution models to predict a woman’s personalized BCaO probability curve (time-dependent probabilities) from both her lifestyle and health history information. We first curated the ATP-BCa dataset that contains both health history and lifestyle information of 18,288 healthy (at the time of recruitment) females who were followed up for their BCaO. We used this ATP-BCa dataset to evaluate the effect of various combinations (*3 × 5 × 9 = 135*) of imputation methods, feature selection methods, and ISD models. We evaluated these combinations with both discrimination and calibration through three evaluation metrics: L1-Hinge loss, Concordance index, and D-Calibration [24]. We demonstrated the importance of L1-Hinge loss for evaluating models that compute personalized BCaO probabilities and explained that it differs from the standard concordance measure; we also show that L1-Hinge loss is often more relevant. Our results showed that the multi-task logistic regression algorithm [42, 43] with MICE imputation policy and multivariate Cox feature selection method, had the lowest L1-Hinge loss among all D-calibrated models and also had a competitive Concordance index, for BCaO prediction. This paper also described the top ten features that were identified as important for predicting BCaO, for ATP-BCa participants. Among the identified 10 important features, 7 of them were actionable lifestyle features that included supplement consumption, social support index, and food nutrient consumption. This paper then suggests ways to motivate these subjects to change those lifestyle features, to increase their number of BCa-free years.

## Supporting information

S1 Text Evaluation metrics. (PDF)

S2 Text Ablation study. (PDF)

S1 Fig Schematic illustration of our breast cancer onset prediction framework. (TIF)

S2 Fig The sideways decile histogram used for the D-Calibration measurements for DeepHit model using MICE imputation and multivariate Cox feature selection method. (TIF)

S3 Fig Visual comparison of the model performance using different finetune epochs with proposed uncensored L1 loss. (TIF)

S1 Table Comparison of the model performance using different finetune epochs with proposed uncensored L1 loss. (PDF)

S2 Table Details of ISD model implementations. (PDF)

S3 Table Comparison of various combinations (3 × 5 × 9 = 135) of imputation methods, feature selection methods, and ISD models. (XLSX)

S4 Table Feature name dictionary. (XLSX)
