# Supplementary material for: Personalized breast cancer onset prediction from lifestyle and health history information
Source: PLoS One. 2022 Dec 19;17(12):e0279174. doi: 10.1371/journal.pone.0279174 (PMC9762602; doi:10.1371/journal.pone.0279174)
Supplement: S1 Text — (PDF) [file pone.0279174.s001.pdf]

Supplementary Materials for

Personalized Breast Cancer Onset Prediction from Lifestyle and Health History Information

Shi-ang Qi, Neeraj Kumar, Jian-Yi Xu, Jaykumar Patel, Sambasivarao Damaraju¶, Grace Shen-Tu¶, Russel Greiner¶*

¶ These authors contributed equally to this work
* Corresponding author. Email: rgreiner@ualberta.ca

This PDF file includes:

Supplementary Text A
References [24]

Supplementary Text

A. Evaluation Metrics

In this section, we describe three performance metrics that we used in the paper:

A.1. L1-Hinge Loss

L1 loss (or mean absolute error) measures the difference between the observed event time and the predicted median event time for an uncensored patient. Although each instance must have a precise event in the traditional regression tasks, many instances are censored in the survival analysis tasks. To incorporate censored patients into the calculation, we calculate the Hinge loss for the whole dataset D as presented by:

$$L1_{hinge}(\{\delta_i, t_i\}_{i \in D}, \{\hat{t}_i\}_{i \in D}) = \frac{1}{|D|} \sum_{i \in D} \delta_i |t_i - \hat{t}_i| + (1 - \delta_i)[t_i - \hat{t}_i]_+$$

where subscript i denotes the i-th patients, δ_i denotes the event/censor indicator for that patient (0 means censored and 1 means uncensored), t_i denotes the event/censor time, and $\hat{t}_i$ denotes the predicted event time (which is the median time of the predicted survival curve).

For an example, the L1-hinge loss for a patient who was censored at the time t_i, whose predicted median survival times was $\hat{t}_i$, is $[t_i - \hat{t}_i]_+$: if t_i is larger than $\hat{t}_i$, this is $t_i - \hat{t}_i$, but if t_i is smaller than $\hat{t}_i$, then this is 0. So, if a model predicts a patient's median survival time was $\hat{t}_i = 3$ years, and this patient was censored at $t_i = 5$ years, then the L1-hinge loss would be $[5 - 3]_+ = 5 - 3 = 2$ years. However, if this patient, instead, was censored at 2 years, the L1-hinge loss would be $[2 - 3]_+ = 0$ years. See *[24]* for details.

A.3. Concordance Index

Concordance index, or C-index, considers all "comparable" pairs, and computes the proportion of these pairs whose predicted order of event matches the true order. Given a dataset $D = \{x_i, \delta_i, t_i\}_{i \in D}$ and an ISD model Θ, the C-index can be defined as:

$$C(\Theta, D) = \frac{\sum_{i:\delta_i=1} \sum_{j:t_i<t_j} \mathbb{1}[\text{Median}(\hat{S}_\Theta(\cdot\,|x_i)) < \text{Median}(\hat{S}_\Theta(\cdot\,|x_j))]}{\sum_{i:\delta_i=1} \sum_{j:t_i<t_j} 1}$$

where $\text{Median}(\hat{S}_\Theta(\cdot\,|x_i))$ denotes the median event time of the ISD curve for the i-th patient, predicted by the model Θ. The indicator function $\mathbb{1}[\cdot]$ returns 1 if the statement inside is true while 0 if the statement is false. The denominator in the above equation defines the "comparable pairs" in the C-index computation. For example, any two uncensored individuals obviously form comparable pairs. However, a censored patient can only form a "comparable" pair with an uncensored patient whose predicted event time (here, median of the subject's BCaO ISD) is less than her censoring time.

A.3. Distribution Calibration

Distribution calibration, aka D-calibration, is a statistical test to evaluate the calibration ability of the ISD models (which produce event probabilities for all future times). For the notation, give a dataset $D = \{x_i, \delta_i, t_i\}_{i \in D}$, an ISD model Θ, and any interval $[a, b] \subset [0, 1]$, let

$$V_{\Theta,\text{D}}([a, b]) = \{[x_i, \delta_i = 1, t_i] \in D | \hat{S}_\Theta(t_i|x_i) \in [a, b]\}$$

be the subset of patients in D whose time-of-event-probability (according to $\hat{S}_{\Theta}(t_i|x_i)$) is in the interval $[a, b]$. This model Θ is considered D-calibrated if the proportion of patients $|V_{\Theta,\mathrm{D}}([a, b])|/|D|$ is statistically similar to the proportion $b - a$. In this paper, we use 10 equal-sized bins from [0, 0,1) to [0.9, 1], and then compare the distributions of predicted and observed proportions of events using the Hosmer-Lemeshow test. To incorporate censored patients, Haider et al. proposed a method by "splitting" each censored patient uniformly among the time bins after the censored time *[24]*.

Reference:

[24] Haider H, Hoehn B, Davis S, Greiner R. Effective Ways to Build and Evaluate Individual Survival Distributions. J Mach Learn Res 2020;21:1–63.
