# Supplementary material for: Personalized breast cancer onset prediction from lifestyle and health history information
Source: PLoS One. 2022 Dec 19;17(12):e0279174. doi: 10.1371/journal.pone.0279174 (PMC9762602; doi:10.1371/journal.pone.0279174)
Supplement: S2 Text — (PDF) [file pone.0279174.s002.pdf]

Supplementary Materials for

Personalized Breast Cancer Onset Prediction from Lifestyle and Health History Information

Shi-ang Qi, Neeraj Kumar, Jian-Yi Xu, Jaykumar Patel, Sambasivarao Damaraju¶, Grace Shen-Tu¶, Russel Greiner¶*

¶ These authors contributed equally to this work
* Corresponding author. Email: rgreiner@ualberta.ca

This PDF file includes:

Supplementary Text B

Supplementary Text

B. Ablation Study

In this section, we take the best-performing model (MTLR) from the previous experiment and derive this model with a different type of objective function for training. We proposed to incorporate the target evaluation metric - L1-Hinge loss - into our objective functions and minimize them directly. However, the natural non-differentiable characteristic of L1 poses a challenge for optimization. Instead, we formulate a soft version of L1 loss using a soft membership function name uncensored soft L1 loss. We hypothesize that using our proposed objective function will help the MTLR model train faster and obtain better L1 performance without compromising the C-index score.

The original MTLR can be seen as a series of logistic regression models built on discrete-time intervals to estimate the event happening probability *(42)*. It transforms the short data structure (censor indicator δ and time T) to a long data structure (a status vector $y = (y_1, y_2, \ldots, y_k)$ indicates whether the event happened in each time interval or not, where K is the bin of time), and derive the likelihood function as:

$$L_{nll}(\theta, D) = \sum_{j=\text{all}} \log\left(\sum_{i=1}^{K} \exp\left(\sum_{k=i}^{K} f_k\left(x^{(j)}\right)\right)\right) - \sum_{j=\text{uncensored}} \sum_{k=1}^{K} f_k\left(x^{(j)}\right) \cdot y_k{}^{(j)} - \sum_{j=\text{censored}} \log\left(\sum_{t_k > t^{(j)}} \exp\left(\sum_{k=t_k}^{K} f_k\left(x^{(j)}\right)\right)\right) + c \cdot \|\omega\|_2 \quad (1)$$

where $f_k(x^{(j)})$represents the multi-task probability for j-th individual using logistic regression *(42)* or neural network *(43)* at time interval k, and c represents the scale of the penalty term.

As a popular evaluation metric for regression tasks, L1 loss calculates the difference between the observed event time and the predicted event time. To incorporate with the transformed long data structure of the time-to-event data, the formal L1 loss definition for uncensored individuals can be written as:

$$L_{uncensored} = \sum_{j=\text{uncensored}} \left| \underset{k}{\text{Median}}[1 - S(\cdot\,|x^{(j)})] - \underset{k}{\text{argmax}}(y^{(j)}) \right| \quad (2)$$

where $\text{Median}[\cdot]$ represents the median event time of a given probability curve, as indicated in the intersections of ISD curves and median lines in Fig 1 in the main text. Optimizing this uncensored L1 loss is difficult because of the non-differentiable nature of the $\text{Median}[\cdot]$ operator and the $\text{argmax}(\cdot)$ operator. Therefore, we first resort to the argmax approximation to replace the $\text{Median}[\cdot]$ operator, specifically,

$$\underset{k}{\text{Median}}[1 - S(\cdot\,|x^{(j)})] \approx \underset{k}{\text{argmax}}[\min(0.5, 1 - S(\cdot\,|x^{(j)}))] \quad (3)$$

And then we further approximate the two argmax operators in L1 uncensored loss with the commonly used soft-argmax function:

$$\underset{k}{\text{softargmax}}(a) = \sum_{i=0}^{K} i \cdot \exp(\beta \cdot a_i) \Big/ \sum_{i=0}^{K} \exp(\beta \cdot a_i) \quad (4)$$

The resulting uncensored soft L1 loss can be then represented as:

$$L_{uncensored} = \sum_{j=\text{uncensored}} \left| \underset{k}{\text{softargmax}}\left[\min(0.5, 1 - S(\cdot\,|x^{(j)}))\right] - \underset{k}{\text{softargmax}}(y^{(j)}) \right| \quad (5)$$

We proposed to use this uncensored soft L1 loss together with the likelihood loss for fine-tuning the MTLR model.

The visual and numerical results are demonstrated in S3 Fig and S1 Table. We adapted the trained MTLR model with MICE imputation and multivariate Cox feature selection as the baseline model, and investigate the effect of the proposed objective function when we use it in the finetuning phase. It can be seen as pretraining a model on the whole dataset using Equation (1) and finetuning the model using a specific (uncensored) population using the combination of Equation (1) and (2). As we can see in S3 Fig, L1-Hinge loss gradually decreases with the increase of finetuning epochs, while the C-index hasn't compromised too much. However, one thing to note is that the finetuned models are not calibrated after the 3rd epoch. Therefore, we suggest that this soft L1 uncensored loss can be used to fine-tune the model in a few epochs (depending on the learning rate and D-calibration performance) for better performance.
