# Supplementary figures and images for: Personalized breast cancer onset prediction from lifestyle and health history information
Source: PLoS One. 2022 Dec 19;17(12):e0279174. doi: 10.1371/journal.pone.0279174 (PMC9762602; doi:10.1371/journal.pone.0279174)

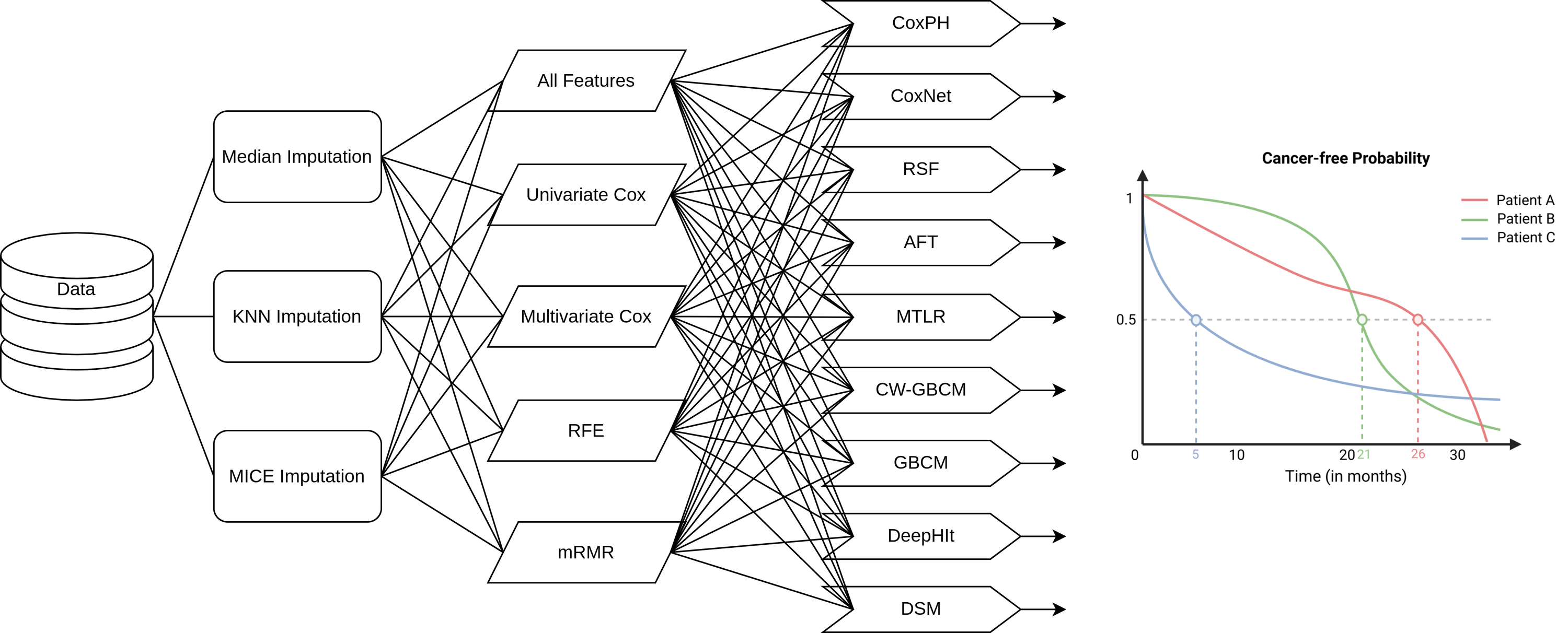

Supplement: S1 Fig — (TIF) [file pone.0279174.s003.tif]

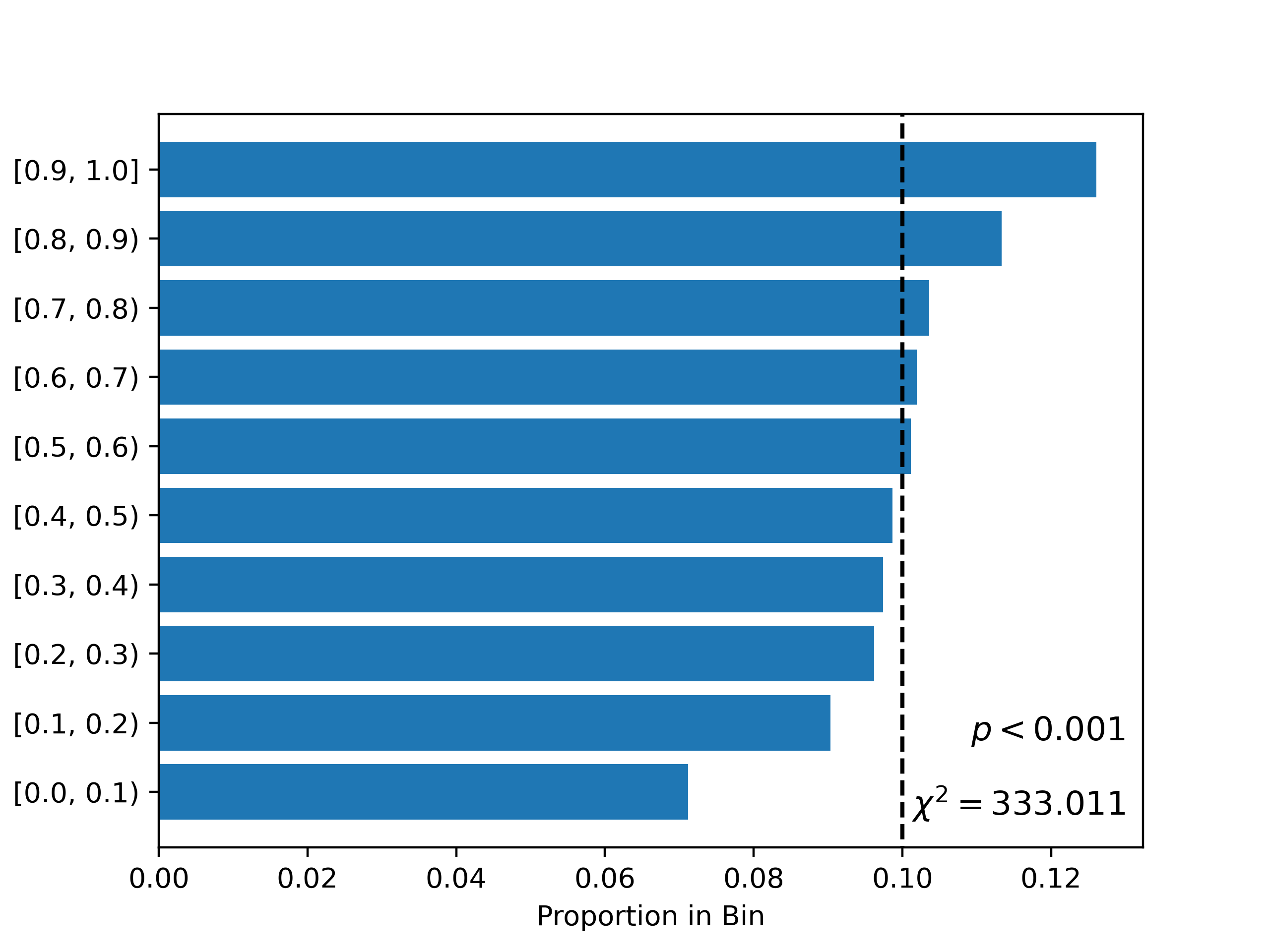

Supplement: S2 Fig — (TIF) [file pone.0279174.s004.tif]

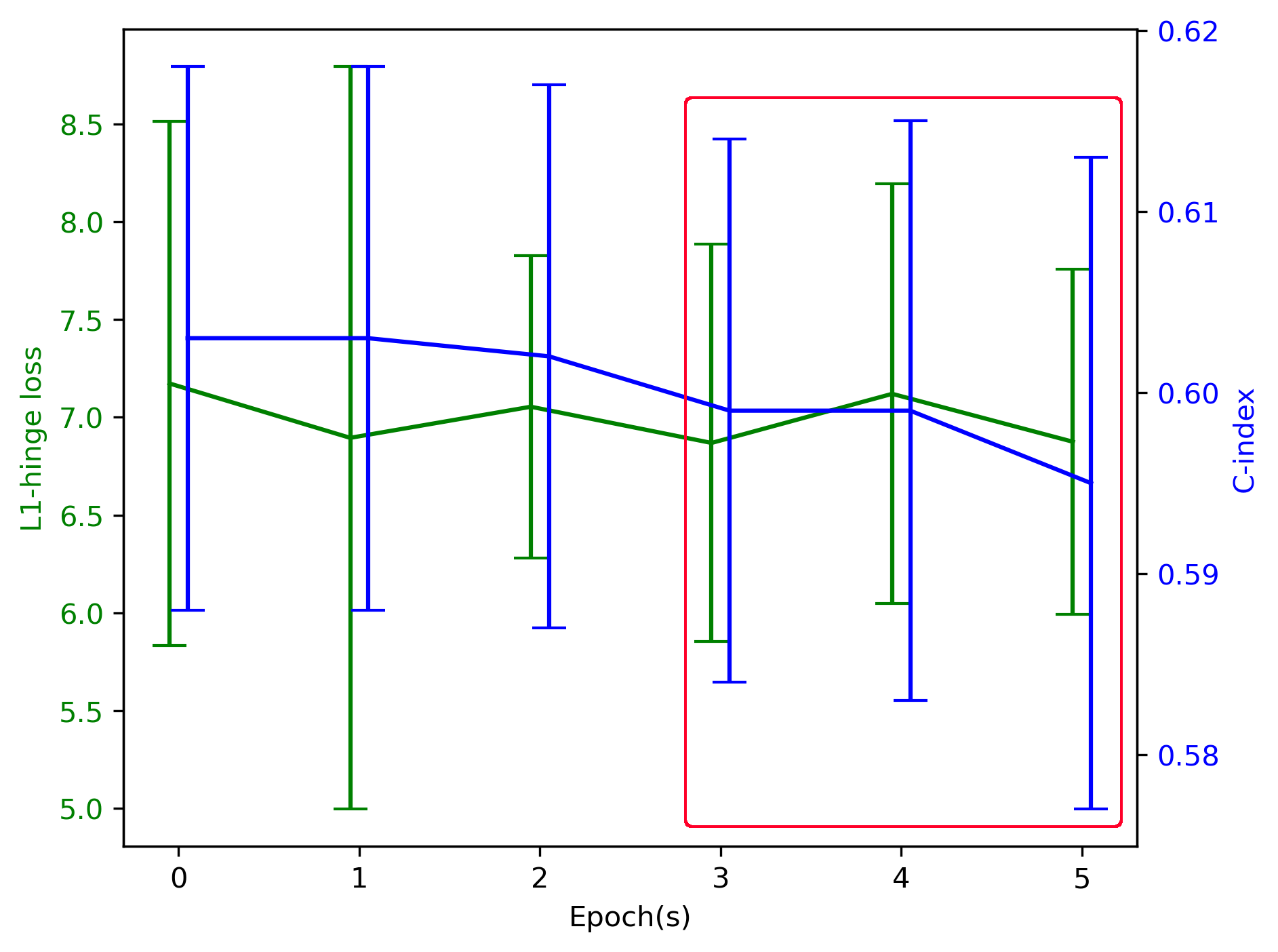

Supplement: S3 Fig — (TIF) [file pone.0279174.s005.tif]
