# Supplementary material for: Personalized breast cancer onset prediction from lifestyle and health history information
Source: PLoS One. 2022 Dec 19;17(12):e0279174. doi: 10.1371/journal.pone.0279174 (PMC9762602; doi:10.1371/journal.pone.0279174)
Supplement: S1 Table — (PDF) [file pone.0279174.s006.pdf]

Supplementary Materials for

Personalized Breast Cancer Onset Prediction from Lifestyle and Health History Information

Shi-ang Qi, Neeraj Kumar, Jian-Yi Xu, Jaykumar Patel, Sambasivarao Damaraju[¶], Grace Shen-Tu[¶], Russel Greiner[¶*]

[¶] These authors contributed equally to this work
[*] Corresponding author. Email: rgreiner@ualberta.ca

This PDF file includes:

Supplementary S1 Table

Supplementary Tables

S1 Table.

Comparison of the model performance using different finetune epochs with proposed uncensored L1 loss. The baseline results are obtained using MTLR with MICE imputation and multivariate Cox feature selection.

Finetune epoch	Finetune learning rate	Finetune loss type	Concordance	L1-Hinge	D-Calibration
0 (Baseline)	N/A	N/A	0.603 ± 0.015	7.173 ± 1.341	0.547
1	0.0001	nll+uncensored L1	0.603 ± 0.015	6.895 ± 1.899	0.376
2	0.0001	nll+uncensored L1	0.602 ± 0.015	7.054 ± 0.772	0.090
3	0.0001	nll+uncensored L1	0.599 ± 0.015	6.869 ± 1.015	0.014
4	0.0001	nll+uncensored L1	0.599 ± 0.016	7.120 ± 1.073	0.024
5	0.0001	nll+uncensored L1	0.595 ± 0.018	6.875 ± 0.884	0.002
