# Supplementary material for: Personalized breast cancer onset prediction from lifestyle and health history information
Source: PLoS One. 2022 Dec 19;17(12):e0279174. doi: 10.1371/journal.pone.0279174 (PMC9762602; doi:10.1371/journal.pone.0279174)
Supplement: S2 Table — (PDF) [file pone.0279174.s007.pdf]

Supplementary Materials for

Personalized Breast Cancer Onset Prediction from Lifestyle and Health History Information

Shi-ang Qi, Neeraj Kumar, Jian-Yi Xu, Jaykumar Patel, Sambasivarao Damaraju¶, Grace Shen-Tu¶, Russel Greiner¶*

¶ These authors contributed equally to this work
* Corresponding author. Email: rgreiner@ualberta.ca

This PDF file includes:

Supplementary S2 Table

Supplementary Tables

S2 Table.
Details of ISD model implementations.

ISD models	Program	Package	Hyper-parameter Field
**CoxPH**	R	fastcox	None
**CoxNet**	R	fastcox	L1 ratio, alpha
**RSF**	R	randomForestSRC	Number of trees, node size
**AFT**	R	survival	None
**MTLR**	Python	torchmtlr	Penalty term weights, learning rate
**CW-GBCM**	Python	Scikit-survival	Number of trees
**GBCM**	Python	Scikit-survival	Number of trees
**DeepHit**	Python	pycox	Learning rate
**DSM**	Python	Deep Survival Machines	Number of base models, distribution type, learning rate
